# White Paper on Early Critical Care Services in Low Resource Settings

**DOI:** 10.5334/aogh.3377

**Published:** 2021-11-03

**Authors:** Lia I. Losonczy, Alfred Papali, Sean Kivlehan, Emilie J. Calvello Hynes, Georgina Calderon, Adam Laytin, Vanessa Moll, Ahmed Al Hazmi, Mohammed Alsabri, Diptesh Aryal, Vincent Atua, Torben Becker, Nicole Benzoni, Enrico Dippenaar, Edrist Duneant, Biruk Girma, Naomi George, Preeti Gupta, Michael Jaung, Bonaventure Hollong, Diulu Kabongo, Rebecca J. Kruisselbrink, Dennis Lee, Augusto Maldonado, Jesse May, Maxwell Osei-Ampofo, Yasein Omer Osman, Christian Owoo, Shada A. Rouhani, Hendry Sawe, Daniel Schnorr, Gentle S. Shrestha, Aparajita Sohoni, Menbeu Sultan, Andrea G. Tenner, Hanan Yusuf, Neill K. Adhikari, Srinvas Murthy, Niranjan Kissoon, John Marshall, Abdo Khoury, Abdelouahab Bellou, Lee Wallis, Teri Reynolds

**Affiliations:** 1 George Washington University, US; 2 University of North Carolina, US; 3 Harvard, US; 4 University of Colorado, US; 5 Adventist Health Ukiah Valley, US; 6 Johns Hopkins, US; 7 University Hospital Zurich, CH; 8 University of Maryland, US; 9 Yemeni Association of Emergency Medicine and Disaster, YE; 10 Nepal Mediciti Hospital, NP; 11 Vila Central Hospital, VU; 12 University of Florida, US; 13 Indiana University, US; 14 University of Cape Town, ZA; 15 Hôpital Universitaire de Mirebalais, HT; 16 Addis Ababa University, ET; 17 Brigham and Women’s Hospital, US; 18 georgetown, US; 19 Baylor, US; 20 Yaoundé Central Hospital, CM; 21 McMaster University, CA; 22 Fiji National University, FJ; 23 Universidad San Francisco de Quito, EC; 24 University of Toronto, CA; 25 KATH, GH; 26 Emergency Medicine SMSB, SD; 27 Korle-Bu Teaching Hospital, GH; 28 Partners in Health, US; 29 Muhimbili University, TZ; 30 Medicine Sans Frontier, US; 31 Tribhuvan University, NP; 32 Highland Hospital, US; 33 SPHMMC, ET; 34 UCSF, US; 35 Sunnybrook Health Sciences Centre, CA; 36 BC Children’s Hospital Research Institute, CA; 37 University of British Columbia, CA; 38 EUSEM, FR; 39 Harvard, FR; 40 WHO, CH

## Abstract

This White Paper has been formally accepted for support by the International Federation for Emergency Medicine (IFEM) and by the World Federation of Intensive and Critical Care (WFICC), put forth by a multi-specialty group of intensivists and emergency medicine providers from low- and low-middle-income countries (LMICs) and high-income countries (HiCs) with the aim of 1) defining the current state of caring for the critically ill in low-resource settings (LRS) within LMICs and 2) highlighting policy options and recommendations for improving the system-level delivery of early critical care services in LRS.

LMICs have a high burden of critical illness and worse patient outcomes than HICs, hence, the focus of this White Paper is on the care of critically ill patients in the early stages of presentation in LMIC settings. In such settings, the provision of early critical care is challenged by a fragmented health system, costs, a health care workforce with limited training, and competing healthcare priorities.

Early critical care services are defined as the early interventions that support vital organ function during the initial care provided to the critically ill patient—these interventions can be performed at any point of patient contact and can be delivered across diverse settings in the healthcare system and do not necessitate specialty personnel.

Currently, a single “best” care delivery model likely does not exist in LMICs given the heterogeneity in local context; therefore, objective comparisons of quality, efficiency, and cost-effectiveness between varying models are difficult to establish. While limited, there is data to suggest that caring for the critically ill may be cost effective in LMICs, contrary to a widely held belief. Drawing from locally available resources and context, strengthening early critical care services in LRS will require a multi-faceted approach, including three core pillars: education, research, and policy.

Education initiatives for physicians, nurses, and allied health staff that focus on protocolized emergency response training can bridge the workforce gap in the short-term; however, each country’s current human resources must be evaluated to decide on the duration of training, who should be trained, and using what curriculum.

Understanding the burden of critical Illness, best practices for resuscitation, and appropriate quality metrics for different early critical care services implementation models in LMICs are reliant upon strengthening the regional research capacity, therefore, standard documentation systems should be implemented to allow for registry use and quality improvement.

Policy efforts at a local, national and international level to strengthen early critical care services should focus on funding the building blocks of early critical care services systems and promoting the right to access early critical care regardless of the patient’s geographic or financial barriers. Additionally, national and local policies describing ethical dilemmas involving the withdrawal of life-sustaining care should be developed with broad stakeholder representation based on local cultural beliefs as well as the optimization of limited resources.

## Introduction

The care of critically ill patients remains an essential component of international health systems. For low-resource settings (LRS), particularly in low- and low-middle-income countries (LMICs) with still-maturing health systems, the burden of critical illness is higher [1,2], presentations to the emergency departments for high acuity illness are disproportionately greater [3], patient outcomes are worse [1,2] and the provision of critical care services are challenged by costs, fragmented health care delivery [4], limited healthcare workers with appropriate training [4], and relevancy concerns in the face of competing healthcare priorities [5]. While intensive care units typically require substantial human and physical resources at significant cost [6], intensive care, also known as critical care, fundamentally is the provision of appropriate medical care to the very sick patient at high risk of death [7]. Therefore, critical care does not necessarily require intensive care units or expensive resources.

The manner in which critical care is delivered often depends on the local context due to the heterogeneity of economic, political, educational, and cultural factors between and within geographic regions globally. Furthermore, the available evidence to guide management in LRS remains limited and the research agenda in this space is poorly defined [4]. Given these factors, an international, multiprofessional approach is warranted to ensure that system-level changes are adopted and adapted to optimize the growth, quality and cost-effectiveness of critical care in LRS.

This White Paper, published by the International Federation for Emergency Medicine (IFEM), aims to define the current reality of caring for the critically ill in LRS, highlight policy options and make recommendations for improving the system-level delivery of initial critical care services in LRS. Understanding the unique challenges faced in LRS and recognizing that the care of the critically ill patient occurs in a continuum from the pre-hospital setting to the Emergency Unit (EU) to the inpatient wards, IFEM’s critical care Special Interest Group, a multidisciplinary, multinational panel of intensivists and Emergency Medicine (EM) physicians from high-income countries (HICs) and LMICs developed a series of questions with a particular focus on *early* critical care services in LRS:

1. How are “critical care” and “early critical care services” defined?
2. What is the current reality of early critical care in the LRS context?
3. How should the continuum of early critical care be defined for LRS?
4. How should the impact of early critical care services in LRS be measured?
5. What are the next steps to improve critical care services in LRS?

While the care for the critically ill beyond the early stages of illness is an important topic to address, we have chosen to focus on the initial component or “early” critical care services in this paper as the health services that are required to occur at this time point in patient care are those that must take place in many settings throughout the healthcare system, as described above. Additionally, we recognize that early critical care services may be rendered by clinicians who may not have longitudinal involvement with the patient but whose actions may be determinative of trajectory of illness.

Selected authors answered each of these questions based on available evidence from literature reviews and expert opinion. The discussion centers neither on specific disease processes nor on intensive care unit structure and organization. Rather, the paper focuses on system-level issues and options related to early critical care delivery and enhancement in LRS, in line with IFEM’s stated mission to “advance the growth of high quality emergency medical care” [8].

## Section I

### How Are “Critical Care” and “Early Critical Care Services” Defined?

Unified definitions of many important terms relevant to early critical care services do not yet exist. Variations in terminology across the medical literature can create confusion. For the purposes of this White Paper, we adhere to the definitions noted below. When possible, the definitions are derived from the published literature.

1) *Critical illness*—This paper supports the definition, that critical illness represents any immediately life-threatening disease or injury that, if left untreated, can lead to death [9].

2) *Critical care*—The World Federation of Intensive and Critical Care (WFICC) provides the following consensus definition: [6].

Intensive care, also known as critical care, is a multidisciplinary and interprofessional specialty dedicated to the comprehensive management of patients having, or at risk of developing, acute, life-threatening organ dysfunction. Its common expertise is the pathophysiology and support of organ dysfunction more than the specific management of the diseases responsible for the acute illness; the primary goal of intensive care is to prevent further physiologic deterioration while the underlying disease is treated and resolved.

More simply, critical care is the care given to any patient with critical illness [9]. Crucially, critical care can occur at any point of patient contact—the pre-hospital setting, the Emergency Unit (EU), the general inpatient wards, or the ICU—and does not require a physical ICU space or specially trained personnel to be provided.

3) *Early critical care services*—early critical care services emphasizes vital organ support during the initial medical care provided to the critically ill patient who is still within the dynamic phase (usually within the first 24–72 hours) of critical illness [9]. Early critical care is constrained by patient condition, not time [6] or location within the healthcare system.

4) *Low-middle income country (LMIC)*—The World Bank uses objective economic indices to categorize countries into four primary groups: low-income, lower-middle income, upper-middle income, and high-income economies [10]. This terminology is widely accepted and frequently abbreviated in the medical literature to “low-middle income countries” (LMICs). In this paper, LMIC specifically refers to low- and lower-middle income countries, as defined by the World Bank.

5) *Low-Resource Setting (LRS)*—Neither the WHO, nor the World Bank, nor the United Nations (UN) defines the term “low-resource setting” or its equivalents. Myriad similar phrases used interchangeably in the medical literature include resource-constrained setting, resource-poor setting, developing country, non-industrialized setting, resource-limited setting and austere environment. Each term is vague but generally connotes the idea of a setting with a paucity of material and financial means or human health resources [6]. The term LRS is used throughout this paper to refer to health facilities or health systems in LMICs, acknowledging that LRS exist even in high-income countries.

## Section II

### What is the current reality of critical care in the LRS context?

Epidemiological data indicate that the incidence of critical illness among those living in LRS is rising [11,12,13]. Despite the unique differences in the practice and provision of critical care in LRS compared to resource-rich settings, the vast majority of available data stem from very well-resourced environments. Thus, there exists a sizable gap in knowledge of the realities of contemporary critical care in LRS. Understanding how, where and by whom critical care is provided in LRS is key to informing policy making and resource allocation.

*System components of early critical care services*—Pre-hospital care is crucial in determining patient survival, yet less than 1% of the overall LMIC population has access to dedicated emergency medical services (EMS) [14,15]. The availability of pre-hospital EMS varies greatly between LMICs. Key barriers are the lack of a trained EMS workforce and dedicated vehicles, in addition to the often lengthy distance to a hospital, insufficient early communication with hospital settings or protocols, and limitation of infrastructure. These limitations are often greater in rural areas where a large portion of the population of LMICs live [14,15]. Layperson training on basic life-saving skills is feasible and may help to improve survival of critically ill patients in the pre-hospital setting [15,16,17].

A single “best” care delivery model likely does not exist because optimization depends heavily on the local context. Whereas there is a relatively rigid care pathway in resource rich settings, starting with pre-hospital care and continuing to the EU, the ICU, and then the medical ward, the heterogeneity and fragmentation characteristic of still-maturing health systems in LRS result in a panoply of care pathways. Given this heterogeneity, objective comparisons of quality, efficiency and cost-effectiveness between varying models are difficult. Several common models are presented here; however, this list is not comprehensive. It is important to note that these models are not mutually exclusive, and multiple components may exist within the same health care system.

The *ICU* model is perhaps the most well-recognized. The WFICC developed a three-tier model for adult ICUs with different levels recognizing the variation of resource availability [6]. Level 3 (tertiary) ICUs have extensive resources, the ability to provide comprehensive critical care, and are often part of academic centers. Level 2 (secondary) and level 1 (primary) ICUs have progressively fewer services but can provide early critical care services with agreements to transfer patients to a level 3 ICU, as needed or if available. The WHO mandates that physical ICU space be present in hospitals providing surgical services [18]. Post-operative patients, who formed the majority of critical care admissions in five sub-Saharan countries in a 2007 study, were found to have the greatest survival benefit from ICU admission, indicating the importance of critical care for post-operative patients in LRS [19]. Despite the substantial need for pediatric critical care in LRS, few specialized pediatric ICUs exist. In most LRS, there is an absence of formal training specific to this patient population and most pediatric critical care is delivered in a mixed adult and pediatric ICUs [20,21]. Even when faced with equipment constraints, critical care services can potentially be delivered at small, rural hospitals. One hospital in Uganda effectively managed an eight-bed ICU with one ventilator, donated central lines, and only minimal blood products by focusing on achievable outcomes with the available resources and by limiting nurse patient ratios to 1:4 [19].

The *integrative hospital* model proposes that patients requiring critical care be identified at triage and taken directly to a specific physical location within the hospital that is not necessarily an intensive care unit with advanced equipment but is still a dedicated critical care area. Recommendations for this model include resource provision, lower nurse to patient ratios, and clinical guidelines for the critical care area with a focus on simple and inexpensive tools and equipment [22].

The *hospital-wide triage* model proposes implementing tools that allow for the recognition and treatment of critically ill patients regardless of location. This process is supported by provider education and systemic quality improvement efforts with the goal of improving deployment of relatively low-cost interventions and systems, such as oxygen for pneumonia or resuscitation fluids and antibiotics for septic patients [23]. This model is more forthright regarding the concept of early critical care services, recognizing that the need to care for critically ill patients arises throughout the health care system. Dr. Baker outlines a version of this model in his description of it as Essential Emergency and Critical Care (EECC), defined as “the care that all critically ill patients should receive in all hospitals in the world,” focusing on the most fundamental location-independent, pragmatic and low-cost interventions that can be brought to the patient [24]. Through decentralized triage and early warning scores, EECC prioritizes identification, continued observation, assessment, and treatment of critical illness. Targeted implementation of certain key critical care functions may provide achievable improvement in outcomes in LRS [24].

*Workforce components of early critical care services*—Given the dearth of ICUs in LRS globally [23], a large proportion of early critical care services is provided outside of the ICU by non-intensivists and even non-physicians [25]. Anesthesiologists who may not be trained in critical care provide this care in many settings, particularly for post-operative critical illness [26] but EM providers play a particularly important role, where they exist. While over one dozen LMIC-specific EM physician training programs have been developed in recent years [27], many hospitals in LRS lack specialty-trained EM providers [28,29]. Specialty EM training varies widely across LRS. Such training is associated with reduced mortality [30]. Some of these EM training programs provide focused education on critical care delivery [31], but the extent and impact of such education is not known.

Formal critical care-focused training is increasingly available in LRS [32,33], but these programs are uncommon. While the bulk of formal critical care training emphasizes physicians, some programs underscore the important contribution of non-physician providers [34,35]. Insufficient staffing has been identified as one of the largest barriers to providing safe and effective critical care in LRS [36]. “Brain drain” can play a significant role in exacerbating the local availability of specialist clinicians capable of providing critical care [37,38,39]. Task-shifting, or the training of non-physician providers to fulfill roles traditionally performed by physicians, is proposed as a practical solution to address this physician shortage in LRS [35].

Several initiatives have focused on providing protocolized emergency response training in order to help bridge the gap of specialized formal training. These initiatives include the WHO’s Emergency Triage Assessment and Treatment (ETAT) training for pediatric care [40] and Basic Emergency Care (BEC) for frontline healthcare providers who manage acute illness and injury with limited resources [41], the American Academy of Family Physicians’ Advanced Life Support in Obstetrics [42], and India’s National Trauma Management Course [25].

While multidisciplinary nature of critical care mandates that an entire care team assist the primary clinician, there is a worldwide shortage of trained nurses and other ancillary staff that play essential roles in critical care delivery, as well as a paucity of data describing the extent and impact of this problem in LRS [43]. Nurses with competencies in critical care are generally rare in LRS, but several models have been described to address this problem [44,45]. Similarly, up to 44% of patients lacked access to regular physical therapy in one survey of LMIC ICUs [46]. Initiatives are underway in some LMICs to ensure a well-trained critical care physiotherapy workforce [47]. The roles of critical care trained pharmacists, respiratory therapists, and dieticians in LRS are less well-defined, but are important points for future study.

Irrespective of who provides critical care and where it is provided in the care continuum, the imperative is to ensure a knowledgeable and capable care team. The current reality of critical care in the LRS context suggests that innumerable challenges remain at every level, but nascent efforts are underway to address these deficiencies.

## Section III

### What Is the Continuum of Early Critical Care Services in LRS?

*The continuum of care*: The continuum of care refers to the different phases of care within the health system, typically separated by location or personnel, but ideally with seamless transitions between each [46,48,49]. As pointed out in a recent Lancet Global Health Commission on high-quality health systems, timeliness is an essential function of quality in early critical care [50], which should be delivered throughout the continuum of care, regardless of location. Basic resuscitation can and should be initiated at the initial location of patient presentation. This point is especially important within LRS given the dearth of EUs and ICUs [4,28,30,51,52].

Establishing hospital-specific guidelines that take into account the time-sensitivity of critical illness, the resources required to provide it, the availability of trained providers and infrastructure available is essential. Even within resource rich settings, where separate EUs and ICUs are widely available and have been the traditional system for many years, institutions are experimenting with novel models of care, including combined EU-ICUs, or mobile critical care teams that are not location-based within the hospital [53,54,55].

*Transition points within the early critical care services continuum*—Transitions of care frequently occur across the continuum between providers and between/within facilities. These transitions are a critical link between the continuum components and affect patient outcomes, especially when patients are critically ill and communication regarding details of their care are essential [9].

Several high-frequency transition points have been identified that require special attention.

- Prehospital to EU/triage
- EU/triage – ICU/Ward/OR/interfacility transfer
- Ward/OR – ICU/interfacility transfer
- ICU/Ward/OR – Ward/interfacility transfer
- Temporary transfers from EU/ICU for specialized procedure/study (OR, radiology, GI etc.)
- Transfer from Ward/OR – EU when necessary

Structured transition protocols can utilise locally available resources (i.e., medical personnel, equipment and support services) to optimally arrange critical care services to improve patient outcomes and system efficiency to reduce costs. These protocols should acknowledge that the risk-benefit calculation for a particular transition of care in a LRS may differ from a resource rich setting. These models may vary based on location, environment, facilities, infrastructure, community, personnel, equipment and transport options. Integration of innovative methods of communication and mHealth/telemedicine may be a way to help improve care during these transitions if created appropriately [56,57].

Conditions that require ongoing life-sustaining vital organ support often necessitate physical transfer of the patient, either within the facility or to another that can provide the needed level of care. The risk-benefit decision for moving to another locale should be calculated based on the local context, including available human and material resources and the time sensitivity of the condition or disease process. Every effort should be made during initial resuscitation and stabilization to ensure the safest transfer possible. Ideally, all interfacility transport systems should be capable of providing basic resuscitation; however, this is not the reality in many LRS. Appropriate critical care facilities and infrastructure are often separated by distance, thereby increasing the need for effective transport, or for flexible areas of care in front line hospitals that can provide early critical care services.

Continuous patient monitoring must occur at each transition point in the care continuum. Transitions of care should avoid gaps in monitoring and in the level of care provided that can expose the critically ill patient to unnecessary risk, particularly for interfacility transfers. However, some exceptions to this may present themselves as necessary risks in very LRS. Again, this should be determined by the local context.

Critical errors in the care and management of critically ill patients can occur during signout periods [58,59], so structured communication protocols, including face-to-face verbal handoff and transfer of documentation, should be developed for each transition point in the care continuum. Signout protocols should be simple to remember, easy to use and adopted systematically throughout a facility or system. Signout and documentation should include, at a minimum, initial assessment findings, laboratory or radiographic data, interventions performed and response to them, and the reason for entry into a critical care treatment pathway. Verbal face-to-face signout between providers caring for the patient initially and those administering critical care is ideal, but when this is not possible, telephone handoff should occur, along with accurate written documentation. Successful implementation of signout protocols that reduce miscommunication has been demonstrated in LMICs [60]. There are various formats to choose from, for example the WHO Basic Emergency Care course uses the SBAR model (Situation, Background, Assessment, Recommendations) [41].

Transitions between the health system components are high-risk events, particularly for patients requiring critical care. Establishing clear referral pathways, structured handoff protocols, and consistent documentation requirements can improve communication between the many clinicians who care for these complex patients [61,62].

## Section IV

### Measuring Impact – How is the impact of early critical care services in LRS measured?

Currently, there is a dearth of knowledge and consensus about how to best evaluate critical care services and their efficacy, safety, and quality in LMICs [1,2,51,63,64]. While this is true for a range of healthcare fields, some aspects that are unique to critical care services are the need to evaluate access to time sensitive indicators, such as time to access care or time to recognition of critical illness.

Several professional organizations and leaders in the field have proposed methods for evaluating quality critical care services, but their frameworks mostly focus on HICs [65,66,67,68,69]. Although some signal functions and impact assessment techniques cross over from high- to low-resource settings, many do not. For example, while sepsis is frequently encountered in both high- and low-resource settings, the underlying etiology and pathophysiology may differ [70]. Thus, the care based on principles derived in resource rich settings may not be appropriate in LRS [71].

While many specific metrics cannot be translated directly from HICs to LMICs, the broader categories of how to look at care can still apply. National and international critical care organizations have named a range of process indicators primarily within several key areas, including Access (e.g., timely availability of space, efficient transport systems and adequately trained caregivers) [67,68], Safety (ability to provide intensive monitoring, rapid intervention, and treatment, as well as preventing and responding appropriately to patient harm secondary to healthcare provided), and Effectiveness (utilizing evidence-based medical practices, assessment of mortality and morbidity) [66,72,73,74,75,76,77,78,79,80].

It is inherently more challenging for LMICs to fulfill identical metrics to HICs. Staffing shortages, limited equipment and monitoring systems decrease the ability to comply with and measure structure, process and outcome indicators. However, a cornerstone of improving care for the critically ill is identifying ways to quantifiably improve care. While this is largely a work in progress, standardized metrics and process improvement measures can be successful in LMICs.

Examples of successful measurements across a range of impacts in LRS for specific conditions are listed below.

- Vertical impact: Early sepsis management in Uganda using a care bundle by a dedicated medical officer led to a significant improvement in mortality [81].
- Horizontal impact: A multi-center initiative to implement sepsis bundles in public hospitals in Brazil found that a decreased time to sepsis diagnosis was associated with reduced hospital mortality [82,83].
- Process measure analysis: The implementation of a sepsis treatment bundle in Haiti significantly improved process measures, such as sepsis diagnosis and recognition [84].
- Alternate case definitions: Traditional diagnostic criteria for many conditions may rely on tests that are costly or not readily available in LRS. The Kigali Modification of the Berlin Criteria for the diagnosis of acute respiratory distress syndrome (ARDS) uses SpO_2_ values and lung ultrasound as alternative diagnostic modalities, allowing for a more accurate determination of the incidence of ARDS in LRS [85].

Another important area to evaluate is cost effectiveness. A myth often believed is that critical care services are a highly expensive luxury that primarily address the needs of the chronically ill or those with limited life expectancy. However, in LMICs critical care is often short-term and life-saving in response to an acute illness or injury, frequently of otherwise young and healthy individuals, and has an immense potential to replace quality of life years lost. Additionally, many of these life-saving early critical care interventions, such as IV fluids or physiologic monitoring, or potentially cost effective prehospital transport, are not limited to the expensive pieces of equipment traditionally associated with HIC intensive care units [86]. What data we do have suggests that early critical care services may likely be highly cost effective for this reason [87,88]. However, we need further data and defined methods for analyzing this.

## Section V

### Recommendations – What are the next steps to improve critical care services in LRS?

Strengthening early critical care services in LRS will require a multi-faceted approach, including three core pillars: education, research, and policy. This foundation offers a starting point for governments, NGOs, and medical professionals to evaluate their current systems, plan interventions and measure impact.

*Education*: Human resource development is essential to early critical care services, especially in LRS where human resource shortages are common [89]. Most medical schools in LRS do not include early critical care services in their curriculum, and there are few education and training programs dedicated to early critical care services in LRS [90,91,92]. When planning educational programs to improve human resource capacity, each country’s current human resources must be evaluated to decide on the duration of training, who should be trained, and using what curriculum. Emergency medicine and/or critical care residencies and fellowships in LRS last one to six years [31,72,93,94,95,96]. Specialty and subspecialty training within these fields should be a long-term goal for physicians in LMICs. However, even with the implementation of new formal physician training pathways, these will still likely only create a small group of physicians qualified to manage critically ill patients, and the length of training and small number of graduates are not sufficient to meet human resource needs and so we must look outside traditional training pathways.

Short-term training periods that focus on high-yield topics and the initial approach to a patient rather than broad critical reasoning or a refined skill set are one option. While short-term programs alone cannot fulfill long-term health system needs, they represent a starting point for training in low-resource settings. These include the recently released WHO Basic Emergency Care course, a week-long open source training program that covers the initial resuscitation and stabilization of several critical presentations [97]. Medium-term programs, similar to the two-year programs, train-the-trainer mid-level Emergency Care Practitioner Program in Uganda [93,96], represent a middle ground. A combination of multiple training programs and durations may be needed to improve short and long-term capacity throughout a country.

Early critical care services require a team-based approach, often involving a broad variety of providers and staff across a range of locations. Each cadre of providers requires early critical care training relevant to their roles in the health system. Nurses deliver a large portion of the care in many countries and their training should be prioritized and perhaps include education geared towards respiratory care in order to strengthen capacity. For countries with sufficient capacity to train physician and nurse specialists, consideration should be given to training dedicated critical care specialists while also incorporating early critical care training into the curriculum of other specialties such as emergency physicians, surgeons, OB/GYNs, pediatricians and internal medicine physicians. Since patients can become critically ill at any time in any context, confining early critical care education to a small group of future critical care specialists without also including it in other training programs may hinder the identification and management of these patients.

Training outside of traditional health structures should also be considered: The effectiveness of training lay people in basic lifesaving skills they can use in scenarios involving injuries, births, and even cardiac arrests have been demonstrated in several studies [17,98,99]. While the use of traditional healers may pose a barrier to timely access to care [100], there may be an opportunity to think creatively on how to engage and incorporate their care into the continuum.

The SARS-CoV-2 pandemic has highlighted how technology can be utilized to bring communication and education in ways not traditionally accomplished. Tele-education for critical care is another area that may well be worth exploring further. Limited studies have shown even weekly tele ICU education in a LRS led to decreased mortality, length of ICU stay, and significant cost savings [101].

Important considerations for medical education programs include:

- Ensuring the content of each education program is relevant to the local disease burden and resources available.
- Including structured initial and continuing education aspects, which will relieve the impractical requirement for frequent recertifications [95,102,103,104]; combining formal or classroom training with clinical mentorship.
- Promoting systems to adapt clinical operations as human resource capacity develops, for example by adding additional even basic technologies ranging from pulse oximetry to ultrasound to positive pressure ventilation.
- Wherever possible, curricula should be open source and shared so new programs can adapt existing materials rather than redevelop the same ones.
- System of credentialing by local regulatory bodies is also important for health care workers training to be recognized in other settings.

Within each setting, consider who can best administer and teach educational programs. If local expertise is not available, international paid or volunteer clinician teachers may be necessary. Even in these cases, programs should still be locally driven: care should be taken to ensure visiting clinicians work with a local partner, are appropriately oriented to the local setting and are supervised within existing local structures. If initial training requires regional or international specialists, train-the-trainer models should be prioritized early to ensure sustainability and scalability of educational interventions.

*Research*: Countries must understand the burden of critical disease, best practices for resuscitation, and effectiveness of different early critical care services implementation models. Better evidence is needed to strengthen early critical care clinical services, direct trainings, evaluate implementation models, and understand the costs of early critical care. Development of information systems should be a goal for the implementation of research.

Human resource limitations, methods of documentation, and the fact that early critical care services in LRS is delivered across a heterogeneous network of facilities – including clinics, outpatient departments, formal EDs, and inpatient units – all contribute to the lack of reliable data [105,106,107,108]. The cost of both conducting research as well as being able to publish and reference literature is also a barrier. This baseline limited understanding of the early critical care disease burden, combined with the lack of consensus on the essential components of early critical care and key analytic elements to evaluate early critical care [105,106,107,108], make it hard to prioritize research or assess different implementation frameworks.

An important next step is having a coalition of experts in emergency and critical care services in LRS in LMICs come together for a priority setting exercise. Ideally this will involve all relevant stakeholders including but not limited to health care providers, policy makers, in addition to academicians. National and international consensus should be reached on the essential components for early critical care systems and quality monitoring to help standardize and carry out outcomes and implementation research. Until then, previously proposed priorities for emergency and critical care research could be adapted. Among others, these include [105,106,107,108]:

- Document and estimate the burden of critical disease through national surveillance systems and analytic models.
- Improve local research infrastructure by supporting qualitative and comparative research and reviewing existing methods of documentation.
- Evaluate access to critical care services by determining where care is provided and by whom, conduct qualitative research examining the settings where this care is being delivered, and evaluate barriers that people face when seeking access to this care.
- Document, classify, and evaluate the range of interventions designed to improve early critical care systems, identify the most relevant outcomes, and create frameworks to evaluate common categories of initiatives.
- Identify health metrics to estimate the burden of preventable disease and its association with a lack of adequate early critical care services. Identify and classify outcome measures needed to evaluate interventions.
- Develop advanced modeling to assess the financial and health effects of implementations of proposed acute care initiatives.

Health system investments are required to address current barriers to research in LRS, including methods of documentation, available infrastructure, and training in research methodology [105,106,107,108].

- Research projects led by HIC researchers should include local capacity building and health systems strengthening.
- Vertical disease-specific research projects should not be ignored, as common diseases in LMICs, including malaria, pneumonia, gastroenteritis, meningitis, tuberculosis, human immunodeficiency virus infection, and bacterial sepsis, contribute to the burden of critical illness [64,109,110].
- Quality improvement projects can be integrated as part of training models to incorporate research methodology as well as real-time improvement in care [111].

*Policy*: Policy needs to be developed and advanced at local, national, and international levels. At an international level, the need for and importance of improved early critical care services should be explicitly recognized and stated, similar to World Health Assembly resolution 60.22 for emergency care systems [112]. Global consensus around these issues will help national ministries of health and non-governmental organizations advocate for funding to improve early critical care services. Advocacy itself is an important component of next steps, as this is crucial in determining both prioritization of human resources, as well as infrastructural changes. Importantly, the right to access early critical care is essential to providing the Universal health coverage the WHO promotes as a human right, “defined as ensuring that all people have access to needed health services of sufficient quality to be effective while also ensuring that the use of these services does not expose the user to financial hardship [113].” In addition, international agreement on the key functions of an early critical care system will help inform national policies to ensure these functions can be fulfilled. International consensus on quality metrics and proposing policies to meet quality targets will allow benchmarking between countries to evaluate systems. Proposed minimum standards exist for ICUs [6] and should be modified for all settings where early critical care services are delivered.

At a national level, early critical care services expansion plans should be made and then supported through policy. Early critical care services are a key component of integrated health care systems and their implementation, development and maintenance should be included when conducting assessments of national health systems, especially those related to emergency or operative care. Essential to any improvement in care, national policy should include commitments to funding the building blocks of early critical care services systems, including improved human resources, training, and quality programs, and infrastructure and supplies for critical care. Each Ministry of Health needs to choose and support care delivery models realistic to their setting. This may require task shifting if human resources are limited. Shifting tasks from specialists to mid-level providers and generalists can close gaps in care [114]. Training and empowering nurses to make critical care decisions and to educate younger nurses, potentially under the supervision of an experienced advanced practice provider, can expand the pool of able practitioners and ensure sustainability of the practice model [39,64,110]. Enshrining task shifting choices into policy assists with implementation models and ensures legal compliance and legitimacy.

National early critical care services delivery plans must consider what level of critical care services will be available at each level of the health system, and how patients will move between these levels. Efforts to compile and then implement practice-support tools such as protocols, checklists, standard order sets, and supplies can benefit early critical care services [23].

Though often overlooked, national policies describing if/when it is appropriate not to start or to stop life sustaining treatment, for example in the face of irreversible pathology or no capacity for higher level care, can guide providers and maximize use of limited resources.

Within each hospital there should be policies describing and promoting early critical care services, including policies describing where such patients should be cared for and by whom. Policies protecting the right of patients to receive time sensitive early critical care services before payment should also be promoted. For countries where there is significant decentralization of health care delivery and health policy, provincial or local policies on similar topics to those at the national level will support early critical care services implementation.

## Conclusion

The burden of critical illness and injury in LRS is higher and associated with worse patient outcomes than in HICs. Although intensive care units are commonly associated with high costs, early critical care services may be effectively delivered in diverse settings in a cost-effective manner. Early critical care delivery models can vary and should be context dependent, with specific attention to regional human and material resource availability. There is evidence that early critical care services are already being delivered in both formal and informal ways across many settings in LRS by many different types of providers.

We believe that the development and strengthening of early critical care services is most effective when all components of the continuum of care are addressed, particularly identified high-frequency care transition points. Providers caring for patients should be prepared to identify the need for critical care and to deliver some level of early critical care services regardless of their level of training or setting. Structured guidelines and protocols that are locally appropriate and based upon global best practices should be developed to guide early critical care decision making. A continuing focus on education, research, and policy guidance will be key to advancing this agenda and to improve the outcomes of the most critically ill and injured patients.
